# Induction of cytochromes P450 1A1 and 1A2 suppresses formation of DNA adducts by carcinogenic aristolochic acid I in rats *in vivo*

**DOI:** 10.1016/j.tox.2016.01.011

**Published:** 2016-02-17

**Authors:** Helena Dračínská, František Bárta, Kateřina Levová, Alena Hudecová, Michaela Moserová, Heinz H. Schmeiser, Klaus Kopka, Eva Frei, Volker M. Arlt, Marie Stiborová

**Affiliations:** a Department of Biochemistry, Faculty of Science, Charles University in Prague, Prague, Czech Republic; b Division of Radiopharmaceutical Chemistry, German Cancer Research Center (DKFZ), Heidelberg, Germany; c German Cancer Consortium (DKTK), Heidelberg, Germany; d Analytical and Environmental Sciences Division, MRC-PHE Centre for Environment & Health, King’s College London, London, United Kingdom

**Keywords:** AA, aristolochic acid, AAI, aristolochic acid I, AAII, aristolochic acid II, AAIa, aristolochic acid Ia, AAN, aristolochic acid nephropathy, BEN, Balkan endemic nephropathy, bw, body weight, c_T_, cycle threshold, CYP, cytochrome P450, dA-AAI, 7-deoxyadenosine-*N*^6^-yl)aristolactam I, dA-AAII, 7-deoxyadenosine-*N*^6^-yl)aristolactam II, dG-AAI, 7-deoxyguanosin-*N*^2^-yl)aristolactam I, HPLC, high performance liquid chromatography, HUFs, Hupki (human *TP53* knock-in) mouse fibroblasts, MROD, methoxyresorufin *O*-demethylation, NQO1, NAD(P)H:quinone oxidoreductase, POR, P450 oxidoreductase, PEI-cellulose, polyethylenimine-cellulose, RAL, relative adduct labeling, RT-PCR, real-time polymerase chain reaction, r.t., retention time, SD, standard deviation, TLC, thin-layer chromatography, UUC, upper urothelial tract carcinoma, UV–vis, ultraviolet–visible, Aristolochic acid I, Cytochromes P450 1A1 and 1A2, Oxidative detoxification, Reductive activation, DNA adducts

## Abstract

Aristolochic acid I (AAI) is a natural plant alkaloid causing aristolochic acid nephropathy, Balkan endemic nephropathy and their associated urothelial malignancies. One of the most efficient enzymes reductively activating AAI to species forming AAI-DNA adducts is cytosolic NAD(P)H:quinone oxidoreductase 1. AAI is also either reductively activated or oxidatively detoxified to 8-hydroxyaristolochic acid (AAIa) by microsomal cytochrome P450 (CYP) 1A1 and 1A2. Here, we investigated which of these two opposing CYP1A1/2-catalyzed reactions prevails in AAI metabolism *in vivo*. The formation of AAI-DNA adducts was analyzed in liver, kidney and lung of rats treated with AAI, Sudan I, a potent inducer of CYP1A1/2, or AAI after pretreatment with Sudan I. Compared to rats treated with AAI alone, levels of AAI-DNA adducts determined by the ^32^P-postlabeling method were lower in liver, kidney and lung of rats treated with AAI after Sudan I. The induction of CYP1A1/2 by Sudan I increased AAI detoxification to its *O*-demethylated metabolite AAIa, thereby reducing the actual amount of AAI available for reductive activation. This subsequently resulted in lower AAI-DNA adduct levels in the rat *in vivo*. Our results demonstrate that CYP1A1/2-mediated oxidative detoxification of AAI is the predominant role of these enzymes in rats *in vivo*, thereby suppressing levels of AAI-DNA adducts.

## Highlights

• Oxidation and reduction of aristolochic acid I (AAI) dictate its (geno)toxicity *in vivo*.
• Cytochrome P450 (CYP) 1A1 and 1A2 are induced in rats treated with Sudan I and AAI.
• Induced CYP1A enzyme activity resulted in decreased AAI-DNA adduct levels *in vivo*.
• CYP1A1 and 1A2 mainly detoxify AAI and attenuate its genotoxicity *in vivo*.

## 1 Introduction

**Fig. 1:**
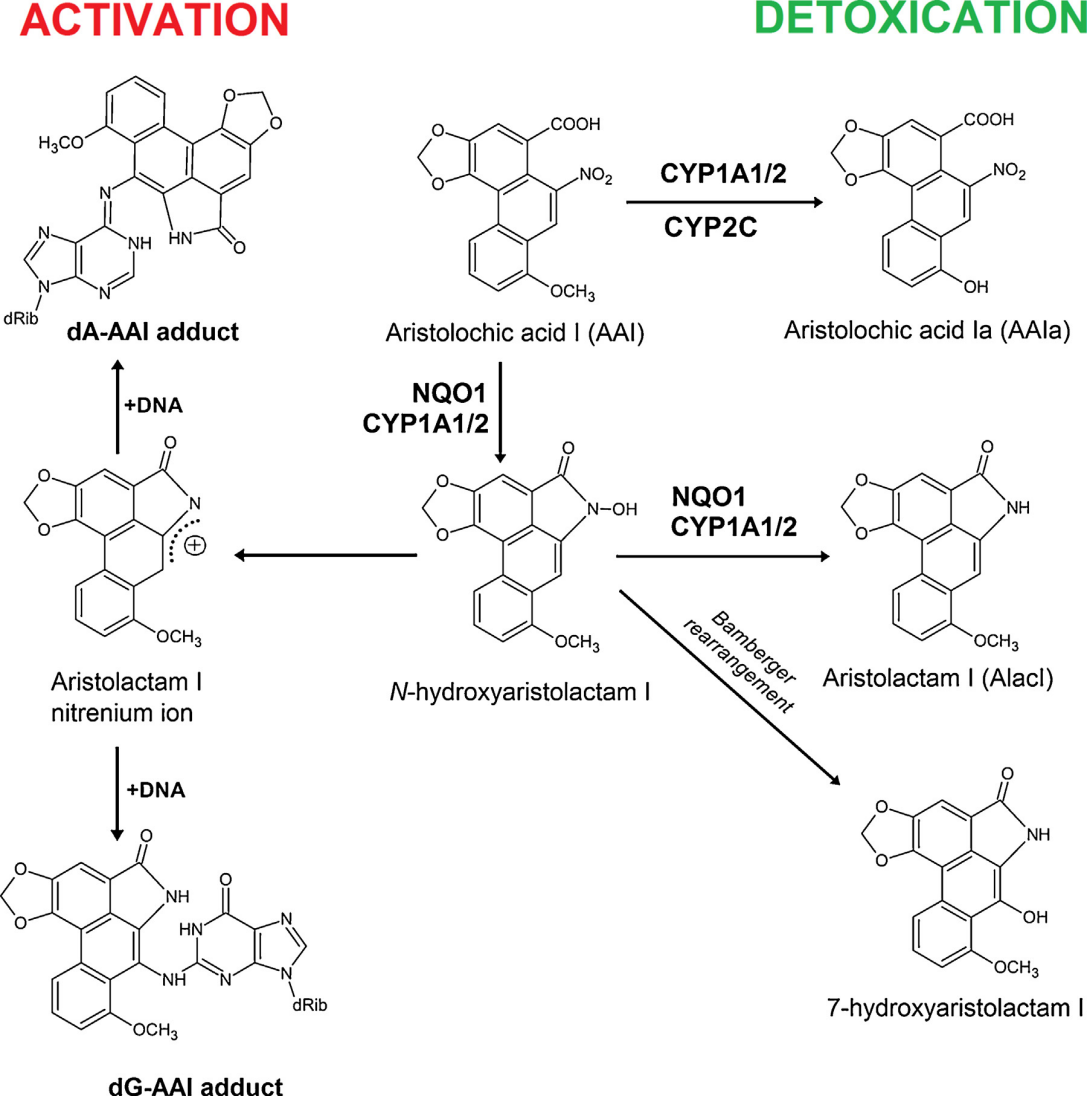
Activation and detoxification pathways of AAI. dA-AAI, 7-(deoxyadenosin-*N*^6^-yl) aristolactam I; dG-AAI, 7-(deoxyguanosin-*N*^2^-yl) aristolactam I; CYP1A1/2, cytochrome P450 1A1 and 1A2; CYP2C9, cytochrome P450 2C9; CYP3A4, cytochrome P450 3A4; NQO1, NAD(P)H:quinone oxidoreductase.

Aristolochic acid (AA) is a herbal drug prepared from plants of the *Aristolochia* genus containing nitrophenanthrene carboxylic acids, of which 8-methoxy-6-nitro-phenanthro-(3,4-*d*)-1,3-dioxolo-5-carboxylic acid (aristolochic acid I, AAI) (Fig. 1) and 6-nitro-phenanthro-(3,4-*d*)-1,3-dioxolo-5-carboxylic acid (AAII) are the predominant components (Arlt et al., 2002b). Over twenty years ago, AA was shown to be the cause of a unique renal disease formerly called Chinese herbs nephropathy, now referred to as aristolochic acid nephropathy (AAN) (for a review, see Arlt et al., 2002b, Schmeiser et al., 2009, Gökmen et al., 2013). AAN is a rapidly progressive renal fibrosis with a high risk for upper urothelial tract carcinoma (UUC) and, subsequently, bladder urothelial carcinoma (Vanherweghem et al., 1993, Nortier et al., 2000, Arlt et al., 2002b, Yun et al., 2012, Gökmen et al., 2013). AA has been classified as a Group I carcinogen by IARC (IARC, 2012). Exposure to AA has also been found to be the cause of a similar type of renal disease, Balkan endemic nephropathy (BEN) and its associated occurrence of urothelial malignancy (Arlt et al., 2007, Grollman et al., 2007). This disease is endemic in certain rural areas of Balkan countries near the tributaries of the Danube river (Schmeiser et al., 2012).

Characteristic AA-DNA adducts in renal tissue of AAN and BEN patients are biomarkers of exposure to AA even long after AA exposure, the 7-(deoxyadenosin-*N*^6^-yl) aristolactam I (dA-AAI) adduct being the most abundant adduct formed and the most persistent (Nortier et al., 2000, Arlt et al., 2002a, Arlt et al., 2002b, Schmeiser et al., 2012, Schmeiser et al., 2014). This deoxyadenosine adduct causes characteristic A–T transversion mutations and such mutations were found in the *TP53* tumour suppressor gene in tumors from AAN and BEN patients (Lord et al., 2004, Grollman et al., 2007) and in immortalized Hupki (human *TP53* knock-in) mouse fibroblasts (HUFs) exposed to AAI (Nedelko et al., 2009). This feature indicates a molecular mechanism of AA-mediated carcinogenesis (Arlt et al., 2007, Kucab et al., 2010). More recently, these A–T transversion mutations were also observed in loci of other genes by whole-genome and exome sequencing analyzing AA-associated UUC and AAI-treated HUFs (Poon et al., 2013, Hoang et al., 2013, Nik-Zainal et al., 2015).

Nitro-reduction of AAI, the compound that is considered as the major cause for AA-mediated development of AAN and BEN, is required to exert its carcinogenic properties (*i.e.* UUC development) (Schmeiser et al., 1996, Schmeiser et al., 2009, Arlt et al., 2002b, Stiborová et al., 2008a, Gökmen et al., 2013). Such nitro-reduction leads to the formation of *N*-hydroxylated aristolactam I which either converts to a reactive cyclic acylnitrenium ion generating DNA adducts or rearranges to 7-hydroxyaristolactam I (Schmeiser et al., 2009). The product of AAI oxidation, 8-hydroxyaristolochic acid I (aristolochic acid Ia, AAIa), is formed by *O*-demethylation of the methoxy group (Fig. 1), and is a detoxification product of this carcinogen. AAIa is excreted either in its free form or conjugated (Chan et al., 2006, Shibutani et al., 2010, Arlt et al., 2011, Stiborová et al., 2012).

Various enzymes are involved in the metabolism of AAI. A variety of studies by us and others have shown that the cytosolic nitroreductase, NAD(P)H:quinone oxidoreductase (NQO1), is the most efficient enzyme activating AAI to DNA adducts (Stiborová et al., 2002a, Stiborová et al., 2003, Stiborová et al., 2008a, Stiborová et al., 2008b, Stiborová et al., 2011a, Stiborová et al., 2013b, Stiborová et al., 2008a, Stiborová et al., 2008b, Martinek et al., 2011, Chen et al., 2011). In human and rodent hepatic microsomes AAI is also activated by cytochrome P450 (CYP) 1A2 and, to a lesser extent, by CYP1A1 and NADPH:cytochrome P450 oxidoreductase (POR) (Stiborová et al., 2001, Stiborová et al., 2005a, Stiborová et al., 2005b, Stiborová et al., 2005a, Stiborová et al., 2005b, Stiborová et al., 2011b, Stiborová et al., 2012, Stiborová et al., 2013b, Stiborová et al., 2005a, Stiborová et al., 2005b, Arlt et al., 2011, Arlt et al., 2015, Levová et al., 2011, Levová et al., 2012, Jerabek et al., 2012) (Fig. 1). However, human and rodent CYP1A1 and 1A2 play a dual role in the metabolism of AAI. Under anaerobic conditions they reductively activate AAI, while under oxidative conditions they are the predominant enzymes catalyzing *O*-demethylation of AAI to AAIa (*i.e.* detoxication) (Stiborová et al., 2001, Stiborová et al., 2005a, Stiborová et al., 2005b, Stiborová et al., 2005a, Stiborová et al., 2005b, Stiborová et al., 2011b, Stiborová et al., 2012, Stiborová et al., 2013b, Stiborová et al., 2005a, Stiborová et al., 2005b, Sistkova et al., 2008, Rosenquist et al., 2010, Arlt et al., 2011, Levová et al., 2011). Beside CYP1A/2, rat and human CYPs of the 2C and 3A subfamilies also oxidize AAI (Sistkova et al., 2008, Rosenquist et al., 2010, Levová et al., 2011, Stiborová et al., 2012, Stiborová et al., 2015a, Stiborová et al., 2015b) (Fig. 1). The CYP-mediated AAI oxidation leads to a decrease in AAI-induced renal injury (Xiao et al., 2008, Xue et al., 2008).

The crucial role of CYP1A1 and 1A2 enzymes in AAI metabolism *in vitro* was unambiguously proven using several systems containing these enzymes [*i.e.* microsomal systems, inhibitors of these enzymes and correlation analyses, recombinant human and rat CYP1A1/2 heterologously expressed in microsomes of insect cells (Supersomes™), purified enzymes reconstituted with POR and other components of the monooxygenase system] (Stiborová et al., 2001, Stiborová et al., 2005a, Stiborová et al., 2005b, Stiborová et al., 2011b, Stiborová et al., 2012, Stiborová et al., 2013b, Stiborová et al., 2005a, Stiborová et al., 2005b, Sistkova et al., 2008, Arlt et al., 2011, Levová et al., 2011). In addition, the importance of CYP1A1 and 1A2 in AAI metabolism has been demonstrated *in vivo* using *Cyp1a1/2*-knock-out (single and double knock-outs) and *CYP1A*-humanized mouse lines (Rosenquist et al., 2010, Arlt et al., 2011, Stiborová et al., 2012, Stiborová et al., 2014a, Stiborová et al., 2014b, Stiborová et al., 2014c). Based on current knowledge we proposed that AAI metabolism by CYP1A1/2 *in vivo* is determined by the binding affinity of AAI to these CYPs, and their enzymatic turnover as well as by the oxygen levels in the organs (Stiborová et al., 2012, Stiborová et al., 2013b, Stiborová et al., 2014a, Stiborová et al., 2014b). Even though several studies considered CYP1A1/2 to be enzymes that detoxify AAI *in vivo* (Xiao et al., 2008, Rosenquist et al., 2010, Arlt et al., 2011, Stiborová et al., 2012, Stiborová et al., 2014a, Stiborová et al., 2014b, Stiborová et al., 2014c), the question which of their two opposing roles in AAI metabolism (AAI detoxification to AAIa *versus* activation of AAI to form AAI-DNA adducts) prevails *in vivo* remains to be answered.

To elucidate the roles of CYP1A this study was performed. AAI was administered to Wistar rats pretreated with Sudan I (1-phenylazo-2-naphthol), a strong inducer of CYP1A1 and CYP1A2 (Refat et al., 2008, Stiborová et al., 2013a), and AAI-DNA adduct levels in target and non-target organs were determined by ^32^P-postlabeling and compared to those in organs of rats treated with AAI only. The amounts of CYP1A1/2 enzymes expressed in rats at transcriptional and translational levels were analyzed by real-time polymerase chain reaction (RT-PCR) and Western blotting, and their activities determined with their marker substrates. The formation of AAIa, the detoxification metabolite of AAI, was analyzed using high performance liquid chromatography (HPLC).

## 2 Materials and methods

### 2.1 Chemicals

NADPH, AAI (sodium salt), Sudan I [1-(phenylazo)-2-hydroxynaphthalene], menadione (2-methyl-1,4-naphthoquinone), cytochrome *c* and calf thymus DNA were from Sigma Chemical Co. (St. Louis, MO, USA). 7-Methoxyresorufin was purchased from Fluka Chemie AG (Buchs, Switzerland). All these and other chemicals were reagent grade or better. Enzymes and chemicals for the ^32^P-postlabeling assay were from sources already described (Stiborová et al., 2005a).

### 2.2 Animal experiments and sample preparation

The study was conducted in accordance with the Regulations for the Care and Use of Laboratory Animals (311/1997, Ministry of Agriculture, Czech Republic), which is in compliance with the Declaration of Helsinki. Animals were purchased from AnLab (Prague, Czech Republic), acclimatized for 5 days and maintained at 22 °C with a 12 h light/dark period. Standardized diet and water were provided *ad libitum*. One group of five weeks old male Wistar rats (∼125–150 g, *n* = 3/group) was treated *i.p.* with a single dose of AAI dissolved in 1% NaHCO_3_ (20 mg/kg body weight, bw), the second group with two doses of Sudan I dissolved in maize oil (*i.p.*, always with 30 mg/kg bw) in two consecutive days, and the third group, where rats were treated *i.p.* with two doses of Sudan I (always with 30 mg/kg bw in two consecutive days) and with AAI (20 mg/kg bw) 24 h after the second dose of Sudan I-treatment. Three control rats received the same volume of both vehicles only. Animals were killed 1 day after the treatment by cervical dislocation. Livers, kidneys and lungs were removed, immediately after sacrifice, frozen in liquid nitrogen and stored at −80 °C. DNA from livers, kidneys and lungs was isolated by extraction with phenol/chloroform (Schmeiser et al., 1996). Total RNA was isolated from another aliquot of frozen organs using Trizol Reagent (Invitrogen, Carlsbad, CA, USA) according to the procedure supplied by the manufacturer. The quality of isolated RNA was verified by horizontal agarose gel electrophoresis, RNA quantity was assessed by UV–vis spectrophotometry on a Carry 300 spectrophotometer (Varian, Palo Alto, CA, USA). Microsomes and cytosols were isolated from the rat tissues by a procedure described previously (Stiborová et al., 2003, Stiborová et al., 2005a). Protein concentration in the microsomal and cytosolic fractions was measured using bicinchoninic acid protein assay (Wiechelman et al., 1988) with bovine serum albumin as a standard. Pooled microsomal and cytosolic samples (*n* = 3 rats/group) were used for analyses. All microsomal and cytosolic samples were free of residual Sudan I, AAI or their metabolites as determined by HPLC (Stiborová et al., 1988, Stiborová et al., 2002b, Stiborová et al., 2005c, Levová et al., 2011).

### 2.3 DNA adduct analysis by ^32^P-postlabeling

The nuclease P1 enrichment version of ^32^P-postlabeling analysis, and thin-layer chromatography (TLC) on polyethylenimine-cellulose (PEI) plates were carried out and DNA adduct levels (RAL, relative adduct labeling) were calculated as described previously (Schmeiser et al., 1996, Schmeiser et al., 2013). AAI-DNA adducts were identified using reference standards as described (Schmeiser et al., 1996).

### 2.4 CYP1A and NQO1 mRNA content in rat livers, kidneys and lungs

RNA samples (1 μg) were reverse transcribed using 200 U of reverse transcriptase per sample with random hexamer primers utilizing RevertAid™ First Strand cDNA Synthesis Kit (MBI Fermentas, Vilnius, Lithuania) according to the manufacturer’s instructions. The prepared cDNA was used for real-time (RT) polymerase chain reaction (PCR) performed in RotorGene 2000 (Corbett Research, Sydney, Australia) under the following cycling conditions: incubation at 50 °C for 2 min and initial denaturation at 95 °C for 10 min, then 50 cycles of denaturation at 95 °C for 15 s and annealing at 60 °C for 1 min, and elongation for 30 s at 72 °C. Gain was set to 7 and fluorescence was acquired after elongation step. The PCR reaction mixtures (20 μl) contained 9 μl cDNA diluted 10-times in Milli-Q ultrapure water (Biocel A10, Millipore, Billerica, MA, USA), 10 μl TaqMan Universal PCR Master Mix (Applied Biosystems, Foster City, CA, USA) and 1 μl TaqMan Gene Expression Assay Mix (commercially available unlabeled PCR primers and FAM™ dye-labelled probe for rat *CYP1A1/2* or *NQO1* as target genes and β*-actin* as reference internal standard gene). Each sample was analysed in two parallel aliquots. Negative controls had the same compositions as samples but cDNA was omitted from the mixture. Data were analyzed by the program RotorGene v6 (Corbett Research, Sydney, Australia) and evaluated by comparative cycle threshold (*c*_T_) method for relative quantitation of gene expression. Cycle thresholds, at which a significant increase in fluorescence signal was detected, were measured for each sample. Then ΔΔ*c*_T_ was evaluated according to following equations: Δ*c*_T_ = *c*_T_ (target) − c_T_ (internal standard), ΔΔ*c*_T_ = Δ*c*_Ttreated_ − Δ*c*_Tcontrol_, where Δ*c*_Ttreated_ is Δ*c*_T_ for treated rats and Δ*c*_Tcontrol_ is Δ*c*_T_ for untreated rats. Δ*c*_T_ is positive if the target is expressed at a lower level than the internal standard (β*-actin*), and negative if expressed at a higher level. The induction of mRNA expression of studied target genes in treated animals was evaluated as 2^−(ΔΔcT)^.

### 2.5 Preparation of antibodies and estimation of CYP1A1, 1A2, and NQO1 protein content in microsomal and cytosolic fractions isolated from rat liver and kidney

The chicken anti-rat CYP1A1, anti-rabbit CYP1A2 and anti-rat NQO1 antibodies were prepared as described previously (Stiborová et al., 2002b, Stiborová et al., 2006). Immunoquantification of microsomal CYP1A1 and 1A2 and cytosolic NQO1 was performed using Western blotting (Stiborová et al., 2006). Rat CYP1A1, rat CYP1A2 and human NQO1 (Sigma) were used to identify the CYP1A1, 1A2 and NQO1 bands, respectively. The antigen-antibody complex was visualized with an alkaline phosphatase-conjugated rabbit anti-chicken IgG antibody and 5-bromo-4-chloro-3-indolylphosphate/nitroblue tetrazolium as dye and bands are expressed as arbitrary units (AU)/mg protein (Stiborová et al., 2002b, Stiborová et al., 2006). Glyceraldehyde phosphate dehydrogenase was used as loading control and detected by its antibody (1:750, Millipore; MA, USA).

### 2.6 NQO1, CYP1A1/2 and 2C6/11 enzyme activity assays

In hepatic, renal and pulmonary cytosols NQO1 activity was measured using menadione (2-methyl-1,4-naphthoquinone) as a substrate; the assay was improved by the addition of cytochrome *c* and NQO1 activity expressed as nmol cytochrome *c* reduced (Levová et al., 2011, Levová et al., 2012). Microsomal samples were characterized for specific CYP1A1 and 1A2 activities: Sudan I hydroxylation to 4′-hydroxy-, 6-hydroxy-, and 4′,6-dihydroxy-Sudan I (CYP1A1) (Stiborová et al., 1988, Stiborová et al., 2002b, Stiborová et al., 2005c) and methoxyresorufin *O*-demethylation (MROD) (CYP1A2) (Burke et al., 1994). Hepatic microsomal samples were also characterized for specific CYP2C6 and 2C11 activities with their marker substrates determining diclofenac 4′-hydroxylation and testosterone 16α-hydroxylation, respectively (Kobayashi et al., 2002, Yamazaki et al., 2006).

### 2.7 Microsomal incubations to study AAI demethylation

Incubation mixtures contained 100 mM potassium phosphate buffer (pH 7.4), 1 mM NADPH, 1 mg rat hepatic, renal or pulmonary microsomal protein and 10 μM AAI in a final volume of 250 μl and were incubated at 37 °C for 20 min; AAI *O*-demethylation to AAIa was determined to be linear up to 25 min. Control incubations were carried out either (i) without microsomes, (ii) without NADPH or (iii) without AAI. AAI and its metabolite AAIa were separated by reverse-phase HPLC, identified by mass spectrometry and quantified as described previously (Levová et al., 2011). Briefly, HPLC was carried out with an Nucleosil 100-5C_18_, 250 × 4.0 mm, 5 mm (Macherey-Nagel) column, using a linear gradient of acetonitrile (20–60% acetonitrile in 55 min) in 100 mM triethylamonium acetate with a flow rate of 0.6 ml/min. A Dionex HPLC pump P580 with UV/VIS UVD 170S/340S spectrophotometer detector set at 254 nm was used. Peaks were integrated with CHROMELEON™ 6.01 integrator. A peak eluting at retention time (r.t.) 22.7 min was identified as AAIa using mass-spectroscopy analysis (Levová et al., 2011). A typical HPLC chromatogram is shown in Supplementary Fig. 1.

### 2.8 Microsomal and cytosolic formation of AAI-DNA adducts

The de-aerated and nitrogen-purged incubation mixtures, in which microsomes were used to activate AAI contained 50 mM potassium phosphate buffer (pH 7.4), 1 mM NADPH, 1 mg of hepatic or renal microsomal protein, 0.5 mg of calf thymus DNA (2 mM dNp) and 0.5 mM AAI in a final volume of 750 μl. Microsomal incubations were carried out at 37 °C for 60 min; AAI-DNA adduct formation was found to be linear up to 2 h in microsomes (Stiborová et al., 2005a). Control incubations were carried out either (i) without microsomes, (ii) without NADPH, (iii) without DNA or (iv) without AAI. After extraction with ethyl acetate, DNA was isolated from the residual water phase as described above (Stiborová et al., 2005a, Stiborová et al., 2011a, Stiborová et al., 2012, Arlt et al., 2011).

The de-aerated and nitrogen-purged incubation mixtures, in which cytosols were used to activate AAI contained 50 mM Tris–HCl buffer (pH 7.4), 0.2% Tween 20, 1 mM NADPH, 1 mg rat hepatic or renal cytosolic protein, 0.5 mg calf thymus DNA (2 mM dNp) and 0.5 mM AAI in a final volume of 750 μl. Incubations with cytosols were performed at 37 °C for 60 min; AAI-derived DNA adduct formation was found to be linear up to 2 h (Stiborová et al., 2003). Control incubations were performed either (i) without cytosol, (ii) without NADPH, (iii) without DNA or (iv) without AAI. After extraction with ethyl acetate DNA was isolated from the residual water phase by the phenol/chloroform extraction method as described above.

### 2.9 Statistical analyses

For statistical data analysis we used Student’s *t*-test. All *P*-values are two-tailed and considered significant at the 0.05 level.

## 3 Results

### 3.1 DNA adduct formation in rats treated with AAI and Sudan I compared to adduct formation in rats treated with AAI alone

**Fig. 2:**
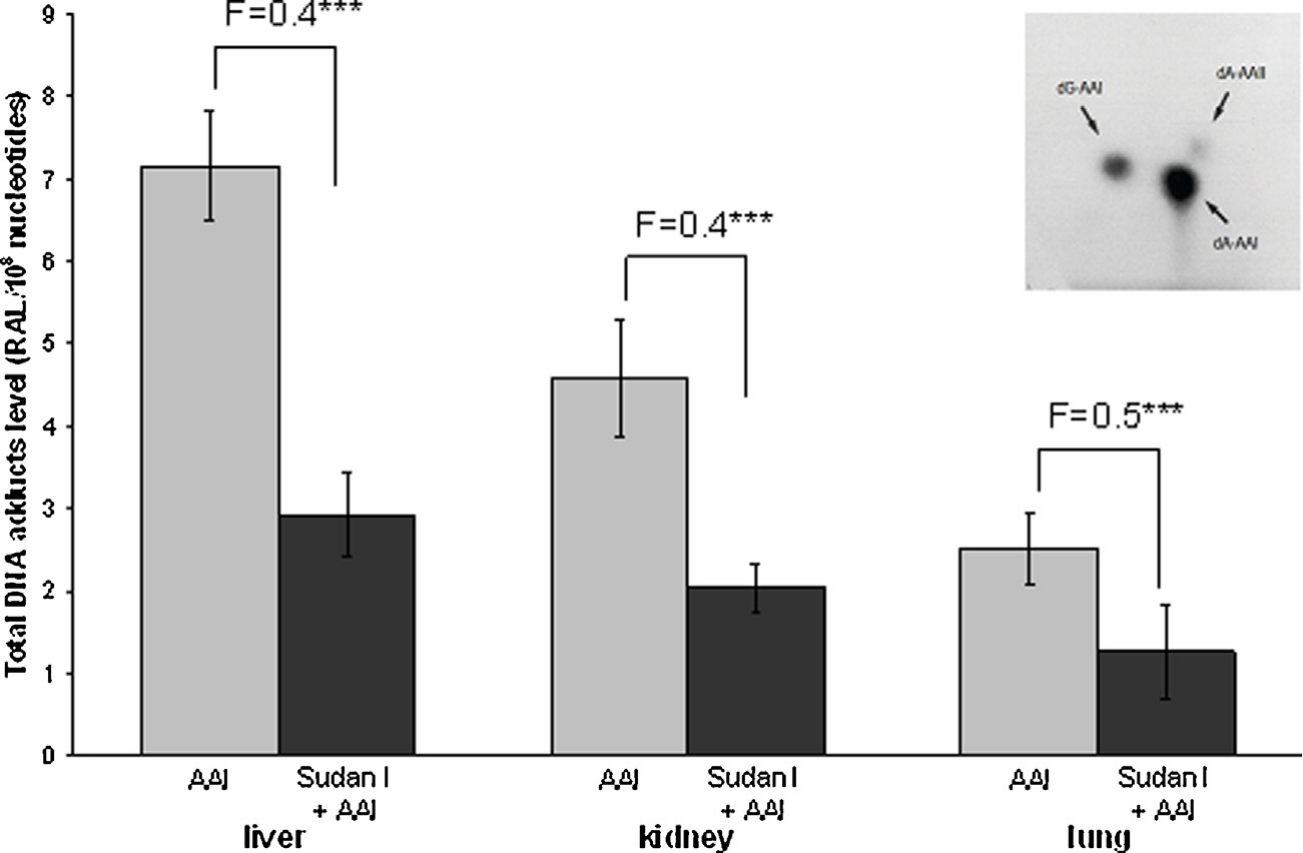
Quantitative TLC ^32^P-postlabeling analysis of AAI-DNA adduct levels in organs of rats treated with AAI, Sudan I or AAI after exposure to Sudan I. Numbers above columns (“F”) indicate fold changes in DNA adduct levels in animals treated with AAI combined with Sudan I compared to animals treated with AAI alone. Values are given as the means ± SD (*n* = 3); each DNA sample was determined by two postlabeled analyses. RAL, relative adduct labeling. Comparison was performed by *t*-test analysis; ***P < 0.001, different from animals treated with AAI alone. Insert: Autoradiographic profile of AAI-DNA adducts formed in liver of rats treated with AAI, determined by the nuclease P1 enrichment version of the ^32^P-postlabeling assay.

AAI-DNA adduct formation was determined by ^32^P-postlabeling in liver, kidney and lung of male Wistar rats treated *i.p.* with AAI, Sudan I, or AAI after pretreatment with Sudan I. Using the nuclease P1 version of ^32^P-postlabeling assay, all liver, kidney and lung samples from rats treated with AAI showed an adduct pattern similar to that found in kidney tissue from AAN and BEN patients (Arlt et al., 2002b, Nortier et al., 2000, Schmeiser et al., 1996, Schmeiser et al., 1997, Schmeiser et al., 2012). As shown in Fig. 2, the adduct pattern consisted of three adduct spots. These spots have been identified as 7-(deoxyguanosin-*N*^2^-yl) aristolactam I (dG-AAI), 7-(deoxyadenosin-*N*^6^-yl) aristolactam I (dA-AAI) and 7-(deoxyadenosin-*N*^6^-yl) aristolactam II (dA-AAII). We have shown previously that the dA-AAII adduct can also be generated from AAI, probably *via* a demethoxylation reaction of AAI or dA-AAI (Stiborová et al., 1994, Schmeiser et al., 1997). No AAI-derived DNA adducts were found in DNA of control rats treated either with vehicle or Sudan I only (data not shown).

Generally, AAI-DNA adduct levels were higher in liver, the organ predominantly responsible for biotransformation of xenobiotics including AAI, as well as kidney, the target organ of AAI genotoxicity (Stiborová et al., 2008a, Stiborová et al., 2008b, Stiborová et al., 2008a, Stiborová et al., 2008b), than in lung (Fig. 2). In all organs of rats treated with AAI after pretreatment with Sudan I, the levels of AAI-DNA adducts were only half of those in rats exposed to AAI alone (Fig. 2 and Supplementary Table 1). Therefore, Sudan I, when administered to rats before their exposure to AAI, shifts the metabolic pathway of AAI that finally leads to a decrease in AAI-DNA adduct levels in all three organs.

Because CYP1A1/2 enzymes both oxidize (*i.e.* detoxify AAI) and reduce (*i.e.* activate AAI to form to AAI-DNA adducts) AAI, their expression might determine the balance between activation and detoxification pathways of AAI (Stiborová et al., 2008a, Stiborová et al., 2008b, Stiborová et al., 2013b, Stiborová et al., 2008a, Stiborová et al., 2008b). Therefore, we investigated whether expression levels of these enzymes influence AAI-DNA adduct formation found *in vivo* (Fig. 2 and Supplementary Table 1).

### 3.2 The effect of AAI treatment with or without Sudan I upon CYP1A1/2 and NQO1 mRNA and protein levels and their enzymatic activities in rat liver, kidney and lung

The effect of exposure to AAI, Sudan I and both compounds on expression of CYP1A1 and 1A2 at the mRNA and protein levels, was examined in liver, kidney and lung.

**Fig. 3:**
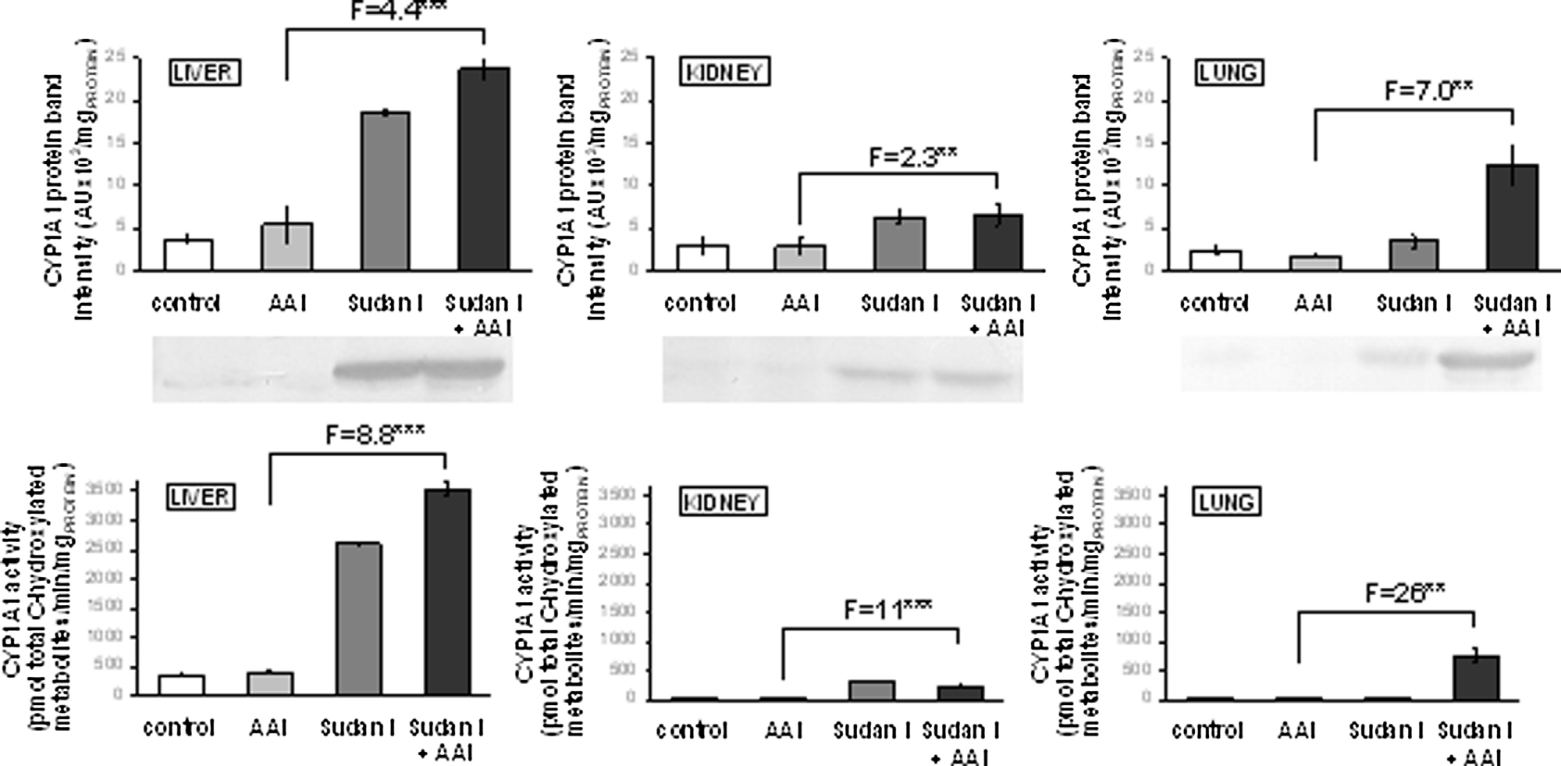
CYP1A1 protein levels (upper panels) in rat microsomes isolated from untreated (control) animals and animals treated with AAI, Sudan I or AAI after exposure to Sudan I. Microsomes isolated from liver, kidney and lung were analyzed by Western blotting in the same blot (insert) and, therefore, can be compared directly. Values are given as the means of arbitrary units (AU per mg protein) ± SD (*n* = 3). CYP1A1 enzyme activity as measured by Sudan I oxidation (nmol total C-hydroxylated Sudan I metabolites/min × mg protein) (lower panels). All values are given as the means ± SD (*n* = 3). Numbers above columns (“F”) indicate fold changes in protein level or enzyme activity in microsomes of rats treated with AAI with Sudan I compared to those with AAI alone. Comparison was performed by *t*-test analysis; **P < 0.01, ***P < 0.001, different from data found in microsomes form rats treated with AAI alone.

The mRNA and protein of CYP1A1 (Table 1 and Fig. 3) were expressed in all organs of control rats. Sudan I oxidation, a marker for CYP1A1 enzyme activity (Stiborová et al., 2002b, Stiborová et al., 2005c), was also detectable in all organs studied, but only very low Sudan I oxidation was measurable in kidney and lung, the organs expressing the lower protein levels of CYP1A1 than liver (Fig. 3).

**Fig. 4:**
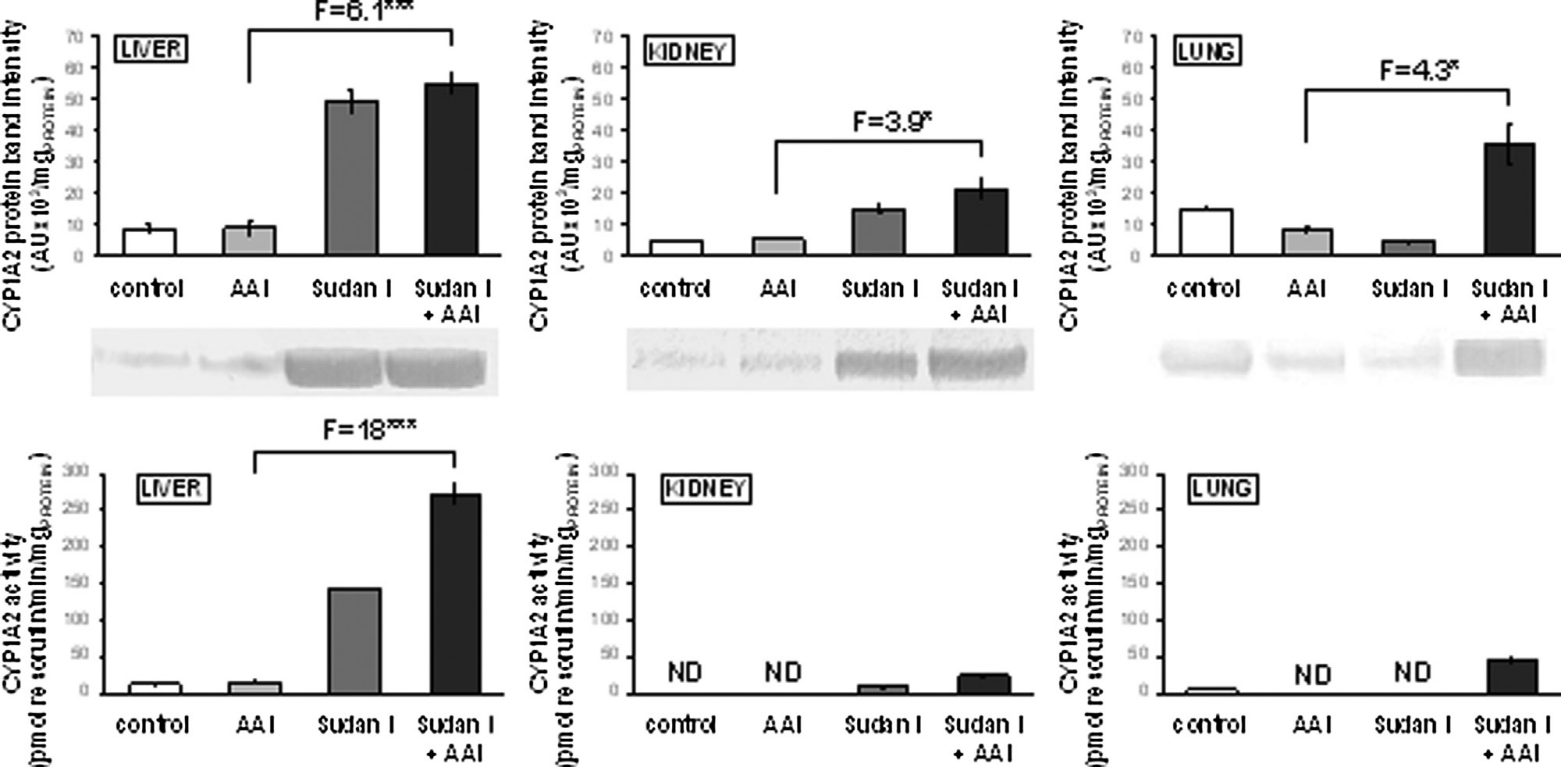
CYP1A2 protein levels (upper panels) in rat microsomes isolated from untreated (control) animals and animals treated with AAI, Sudan I or AAI after exposure to Sudan I. Microsomes isolated from liver, kidney and lung were analyzed by Western blotting in the same blot (insert) and, therefore, can be compared directly. Values are given as the means of arbitrary units (AU per mg protein) ± SD (*n* = 3). CYP1A2 enzyme activity as measured by MROD (pmol resorufin/min × mg protein) (lower panels). All values are given as the means ± SD (*n* = 3). Numbers above columns (“F”) indicate fold changes in protein level or enzyme activity in microsomes of rats treated with AAI with Sudan I compared to those with AAI alone. Comparison was performed by *t*-test analysis; *P < 0.05, ***P < 0.001, different from data found in microsomes form rats treated with AAI alone.

The *CYP1A2* mRNA was expressed mainly in liver (Table 1), whereas the CYP1A2 protein expression levels were higher in liver and lung than in kidney (Fig. 4). In concordance, MROD activity, a marker reaction of CYP1A2, was found in liver and lung, with no activity in kidney (see Fig. 4).

As shown in Table 1, treatment of rats with Sudan I alone or with this compound before exposure to AAI induced expression of *CYP1A1* mRNA in all tested organs. Treatment of rats with AAI alone induced mRNA levels of this CYP only in the liver and lung. The effect of both compounds combined was either the same as of Sudan I alone (lung and kidney) or led to lower mRNA levels in the liver. The most drastic effect was seen in the lung where Sudan I alone or in combination with AAI increased levels of *CYP1A1* mRNA 2900-times as compared to AAI alone (Table 1). Expression of CYP1A1 protein and oxidation of Sudan I, a marker for CYP1A1, were always higher in organs of rats treated with AAI after pretreatment with Sudan I than with AAI alone (Fig. 3).

Expression of mRNA and protein of CYP1A2 was also induced by treatment of rats with AAI, Sudan I or their combined administration (Table 1 and Fig. 4). In liver the mRNA, protein and CYP1A2 enzyme activities ran parallel, in kidney activities were detectable only in microsomes of rats treated with Sudan I or Sudan I combined with AAI. In lung the very high mRNA induction was not reflected in the phenotype; a decrease in amounts of CYP1A2 protein found in lung of rats treated with AAI or Sudan I did not correspond to a 198- or 6170-fold increase in the *CYP1A2* mRNA expression levels (Fig. 4).

The results found confirmed that Sudan I is a strong inducer of CYP1A1/2 in rats and indicate that a combined treatment of rats with Sudan I and AAI leads to even higher enzyme levels than with Sudan I alone.

**Fig. 5:**
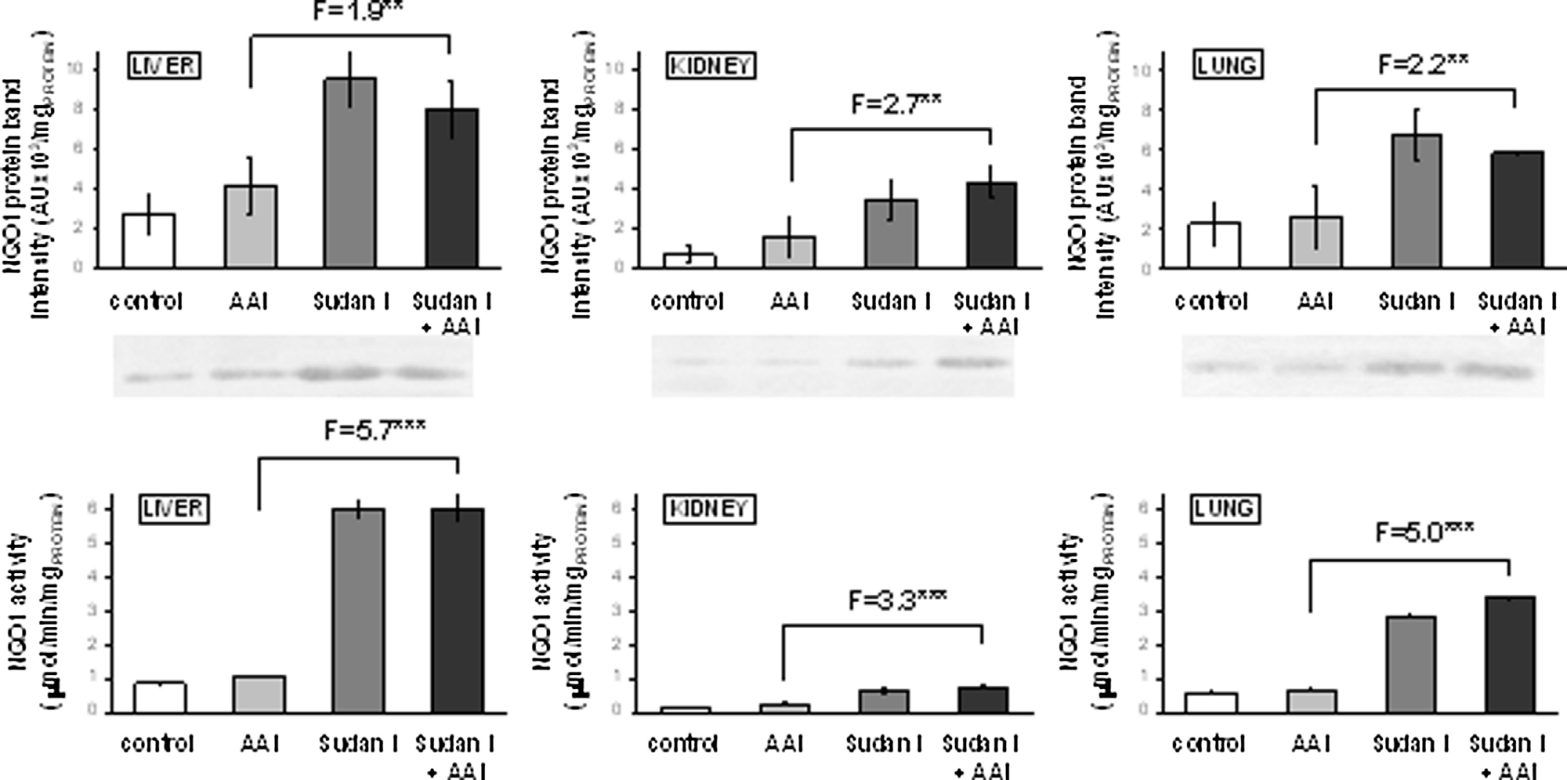
NQO1 protein levels (upper panels) and NQO1 enzyme activity (lower panels) in rat cytosols isolated from untreated (control) animals and animals treated with AAI, Sudan I or AAI after pretreatment with Sudan I. Cytosol isolated from liver, kidney or lung was analyzed by Western blotting in the same blot (insert) and, therefore, can be compared directly. Human recombinant NQO1 was used to identify the rat NQO1 band in rat cytosol (data not shown). Values are given as the means of arbitrary units (AU per mg protein) ± SD (*n* = 3). NQO1 activity in hepatic, renal and pulmonary cytosols was determined using menadione and cytochrome *c* as substrate (expressed as nmol cytochrome *c* reduced/min × mg protein). Numbers above columns (“F”) indicate fold changes in protein level or enzyme activity in cytosols of rats treated with AAI with Sudan I compared to those with AAI alone. Values are given as the means ± SD (*n* = 3). Comparison was performed by *t*-test analysis; **P < 0.01, ***P < 0.001, different from data found in cytosols of rats treated with AAI alone.

Treatment of rats with Sudan I and Sudan I combined with AAI also led to an increased expression of cytosolic NQO1, again at the mRNA, protein and enzyme activity levels in liver, kidney and lung (Table 1 and Fig. 5). Similarly to CYP1A, at the doses used, Sudan I resulted in greater increases at the protein level. Expression of mRNA, protein and enzyme activity of NQO1 measured with menadione as a substrate ran parallel in all three organs and were always higher in organs of rats treated with AAI and Sudan I than in those treated with AAI alone (Fig. 5). However, the efficacy of NQO1 induction by AAI with Sudan I compared to AAI alone was lower than that for CYP1A expression (compare Fig. 3, Fig. 4, Fig. 5). These findings indicate that both compounds administered to rats act as moderate inducers of NQO1.

### 3.3 The effect of treatment of rats with AAI, Sudan I and both agents in combination on oxidation of AAI to AAIa by rat hepatic, renal and pulmonary microsomes

**Fig. 6:**
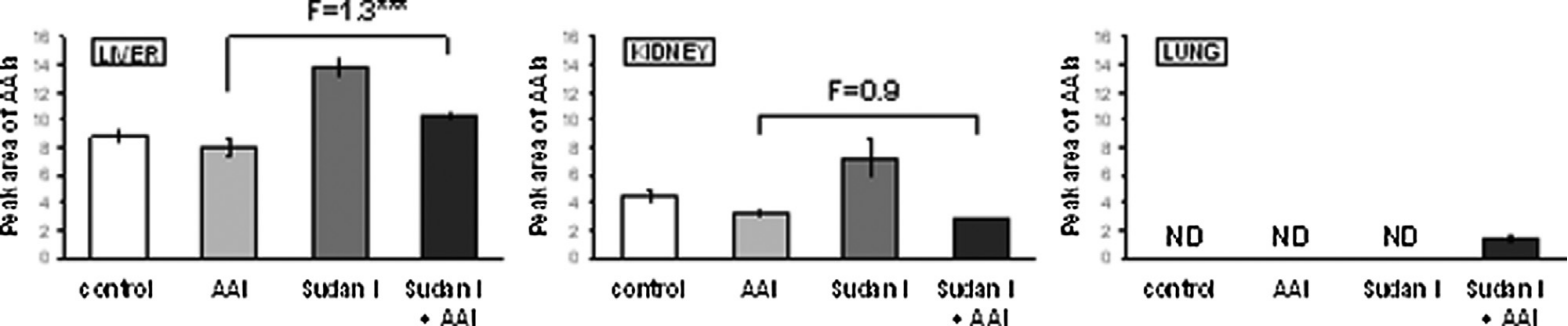
Formation of AAIa (peak area per minute per miligram protein) in rat microsomes isolated from untreated (control) animals and animals treated with AAI, Sudan I or AAI after exposure to Sudan I with AAI as a substrate. All values are given as the means ± SD (*n* = 3). Numbers above columns (“F”) indicate fold changes in AAIa levels in microsomes of rats treated with AAI with Sudan I compared to those with AAI alone. ND, not detected. Comparison was performed by *t*-test analysis; ***P < 0.001, different from data found in microsomes of rats treated with AAI alone.

Since microsomal CYP1A1 and 1A2 detoxify AAI to its oxidative *O*-demethylated metabolite AAIa (Sistkova et al., 2008, Rosenquist et al., 2010, Arlt et al., 2011, Levová et al., 2011, Stiborová et al., 2012, Stiborová et al., 2013b, Stiborová et al., 2014a, Stiborová et al., 2014b, Stiborová et al., 2015b), AAIa formation from AAI was investigated *ex vivo* in hepatic, renal and pulmonary microsomes of all treatment groups. AAIa was formed by liver microsomes from the AAI plus Sudan I group at moderately higher levels as compared to microsomes of rats treated with AAI alone. But in kidney only Sudan I treatment alone increased AAIa formation 1.6-fold (*P* < 0.01), AAI had no effect or even inhibited oxidation of AAI (Fig. 6). In lung the low activity of CYP1A enzymes detectable essentially only in microsomes of rats exposed to both Sudan I and AAI (see the CYP1A1/2 activities determined with their marker substrates shown in Fig. 3, Fig. 4) was confirmed also by formation of AAIa, as AAIa was only detectable at low levels in pulmonary microsomes of this group (Fig. 6). These results indicate that CYP1A1/2 enzymes catalyze AAI demethylation to AAIa in test rat organs, but this activity does not seem to be very effectively induced by Sudan I either alone or in combination with AAI.

**Fig. 7:**
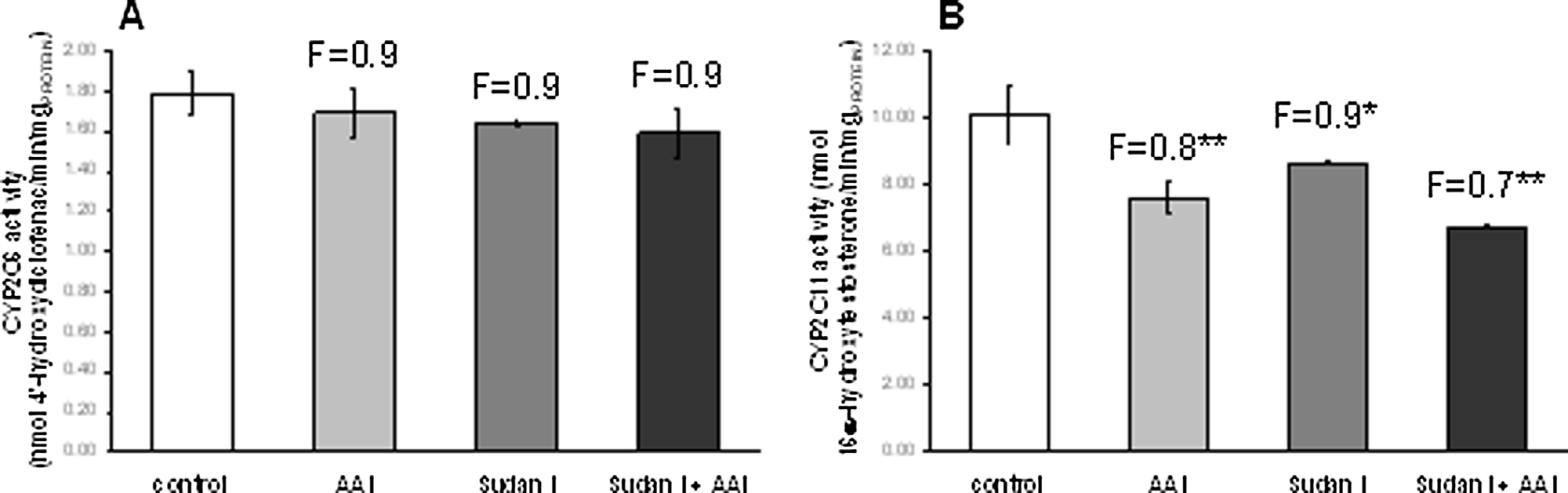
CYP2C6 (A) and CYP2C11 enzyme activities (B) in rat hepatic microsomes. CYP2C6 was measured as diclofenac 4′-hydroxylation (nmol 4′-hydroxydiclofenac/min × mg protein) and CYP2C11 as testosterone 16α-hydroxylation (nmol 16α-hydroxytestosterone/min × mg protein). All values are given as the means ± SD (*n* = 3). Numbers above columns (“F”) indicate fold changes in enzyme activities compared to control. Comparison was performed by *t*-test analysis; ***P < 0.001, different from control.

A probable reason for this observation is that not only CYP1A1/2, but also enzymes of the 2C subfamily, which are highly expressed in the livers of male rats, accounting for approximately 55% of the rat liver CYP complement (Nedelcheva and Gut, 1994), can oxidize AAI. CYP2C11 with ∼50% and CYP2C6 at ∼20% are the main members of the hepatic CYP2C family in rats (Večeřa et al., 2011, Zachařová et al., 2012). Both have been shown to be capable of efficiently oxidizing AAI to AAIa (Levová et al., 2011, Stiborová et al., 2014c, Stiborová et al., 2015a, Stiborová et al., 2015b), and the contribution of the CYP2C enzymes to AAIa formation in rat liver microsomes is more than 4-times higher than that of CYP1A (Stiborová et al., 2015b). Upon induction of CYP1A with Sudan I the relative amount of the CYP2C enzymes in the microsomes will decrease leading to lower CYP2C activity if analyzed based on mg protein, as was the case in our study. To test this, CYP2C activity was also analyzed in hepatic microsomes using diclofenac 4′-hydroxylation for CYP2C6 and testosterone 16α-hydroxylation as a marker for CYP2C11 (Kobayashi et al., 2002, Yamazaki et al., 2006). As shown in Fig. 7 exposure of rats to Sudan I, either with or without AAI, decreased testosterone 16α-hydroxylation activities based on mg protein up to 33% relative to control while diclofenac 4′-hydroxylation was marginally lower. Therefore, decreased relative CYP2C activity could explain why AAIa formation in liver microsomes of rats treated with AAI, Sudan I or with a combination of both compounds did not run parallel to CYP1A induction tested with their marker activities, namely, Sudan I oxidation and MROD.

### 3.4 Microsomal versus cytosolic activation of AAI

In further experiments we investigated whether induction of microsomal CYP1A1/2 and cytosolic NQO1 also influences the reductive activation of AAI to AAI-DNA adducts catalyzed by rat microsomal and cytosolic fractions *ex vivo*. For the investigations we focused on the liver and kidney (target organ for AAI genotoxicity).

**Fig. 8:**
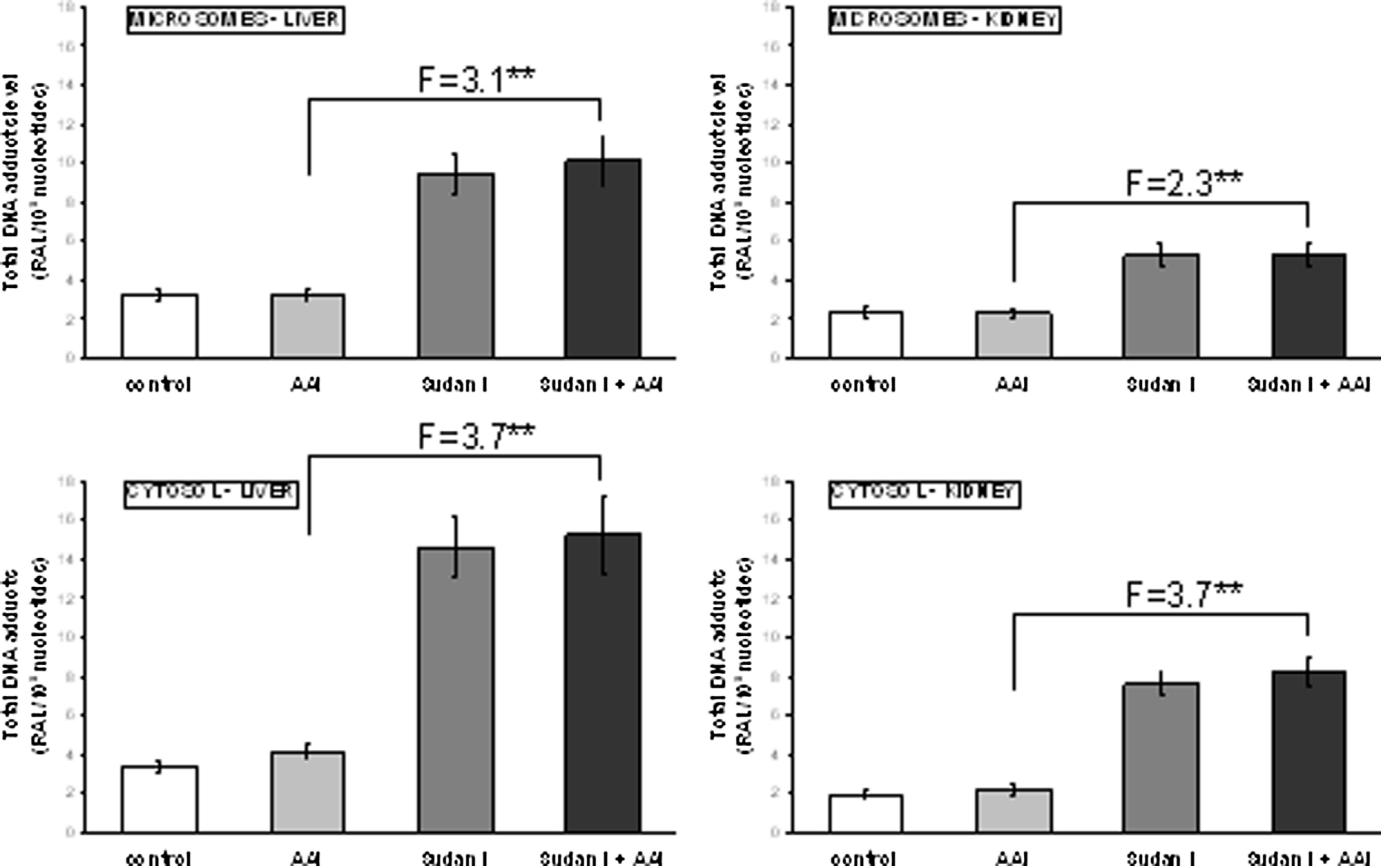
DNA adduct formation *ex vivo* by AAI in rat microsomes (upper panels) and cytosols (lower panels) isolated from liver and kidney of untreated (control) animals and animals treated with AAI, Sudan I or AAI after exposure to Sudan I and incubated with DNA, AAI and NADPH. AAI-DNA adduct formation was determined by ^32^P-postlabeling. Values are given as the means ± SD (*n* = 3); each DNA sample was determined by two postlabeling analyses. RAL, relative adduct labeling. Numbers above columns (“F”) indicate fold changes in AAI-DNA adduct levels in microsomes and cytosols of rats treated with AAI with Sudan I compared to those with AAI alone. Comparison was performed by *t*-test analysis; ***P < 0.001, different from data found with microsomes or cytosols of rats treated with AAI alone.

AAI-DNA adduct formation was analyzed in *ex-vivo* incubations under hypoxic conditions. Incubation mixtures were purged with a stream of nitrogen for 2 minutes before the addition of AAI. AAI was reductively activated by both hepatic and renal microsomes from all treatment groups (Fig. 8). The adduct pattern generated was the same as that found *in vivo* (see Fig. 2). No adducts were observed in control incubations carried out in parallel (data not shown). A significant two to three-fold increase in AAI-DNA adduct formation was seen in incubations of DNA with AAI and hepatic or renal microsomes of rats exposed to Sudan I alone or in combination with AAI (Fig. 8). Overall, the increases in AAI-DNA adduct formation *ex vivo* corresponded to the induction of CYP1A1/2 at protein levels in rats and confirmed the participation of these CYPs in the reductive activation of AAI found previously (Stiborová et al., 2001, Stiborová et al., 2005a, Stiborová et al., 2005b, Stiborová et al., 2012, Stiborová et al., 2014b). The AAI-DNA adduct formation by microsomes under the oxidative (*i.e.* aerobic) conditions was not analyzed in this study. Namely, under these conditions the oxidation of AAI in microsomes (see Fig. 6) should compete with its reduction, which finally result in decreased levels of AAI-DNA adducts. Indeed, as shown in our previous study, an inhibition of AAI-DNA adduct formation occurred in the microsomal system under the aerobic conditions (Schmeiser et al., 1997).

Cytosols, where NQO1 is expressed, were also incubated with AAI, calf thymus DNA and NADPH, the cofactor of NQO1, and analyzed for DNA adduct formation by ^32^P-postlabeling. AAI was activated by hepatic cytosols as evidenced by specific AAI-DNA adduct formation (Fig. 8). No DNA adducts were observed in control incubations carried out in parallel (data not shown). Liver cytosols from rats treated with AAI, Sudan I and AAI after pretreatment with Sudan I produced AAI-DNA adduct levels which were 1.2-, 4.3- and 4.5-fold higher, respectively, relative to cytosols isolated from untreated animals (Fig. 8). The increase in AAI-DNA adduct formation ran parallel to higher NQO1 activity in these cytosols (compare Fig. 5). Renal cytosols isolated from AAI-treated rats, rats treated with Sudan I and rats treated with Sudan I plus AAI led to 1.1-, 3.9- and 4.2-fold higher AAI-DNA adduct levels relative to cytosols from control animals, respectively. Again, the observed adduct levels was consistent with the observed NQO1 enzyme activity (compare Fig. 5, Fig. 8).

## 4 Discussion

CYP1A1 and 1A2 have the dual function to catalyze AAI detoxification to AAIa and the activation of AAI to form AAI-DNA adducts. The aim of this study was to evaluate which of the two opposing functions prevails in an experimental rat model *in vivo*. Here we modulated the expression of CYP1A1/2 by Sudan I treatment which is a strong inducer of these enzymes (Stiborová et al., 2013a, Refat et al., 2008). As a measure of genotoxicity the formation of AAI-DNA adducts was determined. The formation of AAIa was used as a measure for AAI detoxification.

The results of this study demonstrate that AAI-DNA adducts are formed *in vivo* in all organs tested (liver, kidney and lung), both in rats treated with AAI alone or in combination with the inducer Sudan I. These findings suggest that AAI is distributed *via* the blood stream and that these tissues have the metabolic capacity to reductively activate this carcinogen. The levels of AAI-DNA adducts in individual organs therefore depend both on a distribution of AAI to individual organs and on the activities of enzymes catalyzing either its oxidative detoxification or its reductive activation to species forming AAI-DNA adducts. Indeed, our results demonstrate that expression levels of CYP1A enzymes modulate the metabolism of AAI in the rat organs, thereby dictating AAI-DNA adduct formation *in vivo*. Furthermore, it is probable that enhanced clearance of AAI in the liver of induced animals is also altering the levels of AAI-DNA adducts in the kidney.

In our study rats were exposed to AAI for 24 h only to resolve the role of CYP1A1/2 in AAI oxidative or reductive metabolism *in vivo*. We had previously shown the formation of AAI-DNA adducts in liver and kidney 24 h after administration (Pfau et al., 1990, Stiborová et al., 1994, Stiborová et al., 2014c, Arlt et al., 2002b). Therefore, for these experimental purposes and to study the acute effects we used this short exposure, in order to resolve the role of CYP1A1/2 in AAI oxidative or reductive metabolism *in vivo*. Our results indicate that under these conditions AAI genotoxicity (*i.e.* AAI-DNA adduct formation) is reduced after administration of the CYP1A1/2 inducer Sudan I. However, it is important to note that the doses to which humans are exposed to are orders of magnitude lower than the AAI dose administered to rats in this study and its effect at lower but chronic and life-long doses may be different. We found that only half of the AAI-DNA adduct levels were formed in liver, kidney and lung of rats treated with AAI after exposure to Sudan I, than in rats treated with AAI alone (see Fig. 2). These findings demonstrate that induction of CYP1A1 and 1A2 by Sudan I might increase AAI detoxification, leading to lower amounts of AAI available for activation. However, only 1.3-fold higher AAI detoxification (*O*-demethylation activity) was found *ex vivo* in microsomes of treated rats. Previous studies have shown that CYP2C enzymes are also capable in *O*-demethylating AAI (*i.e.* AAI detoxification), and are even more efficient than the CYP1A enzymes to catalyze this reaction in rat liver microsomes (Stiborová et al., 2014c, Stiborová et al., 2015b). CYP2C enzymes constitute about 55% of hepatic CYPs in male rats, Sudan I alone or in combination with AAI induces CYP1A about 4-fold, thereby reducing the relative amount of the other CYP enzymes. In microsomes from CYP1A induced rats, the contribution of CYP2C is therefore lower by a factor of approximately 4 explaining the relatively weak induction of AAIa formation we observed in such microsomes.

The results of the present study fit with the proposed scheme of AAI metabolism (see Fig. 1). If AAI is oxidized to AAIa, lower amounts of AAI are available to be activated by enzymes with nitroreductase activity like NQO1 (for a review, see Stiborová et al., 2008b, Stiborová et al., 2014a, Stiborová et al., 2014b, Stiborová et al., 2014c) which generate cyclic acylnitrenium ions that bind to DNA (*i.e.* DNA adduct formation) (Fig. 1). Our results are in accordance with two previous studies showing that AAI detoxification is lower in *Cyp1a* knockout mice (*i.e. Cyp1a1(-/-)*, *Cyp1a2(-/-)* and *Cyp1a1/2(-/-)* mouse lines) leading to an increase in AAI (geno) toxicity (Rosenquist et al., 2010, Arlt et al., 2011).

Our results of the *ex-vivo* experiments also confirm previous findings (Stiborová et al., 2001, Stiborová et al., 2012, Arlt et al., 2011, Levová et al., 2011) that under hypoxic (anaerobic) conditions, rat hepatic and renal CYP1A enzymes are capable of reducing AAI to species forming DNA adducts. Induction of CYP1A proteins and their enzyme activities correlated with increased AAI-DNA adduct formation *ex vivo* (Fig. 8). Therefore, induction of CYP1A1 and 1A2 leads to both oxidation and reduction of AAI which indicates that in case of hypoxia AAI must act as a ligand of CYP1A heme iron under low pO_2_. Indeed, reduction of AAI as a ligand of heme iron of CYP1A1 and 1A2 could be confirmed by molecular modeling (Jerabek et al., 2012, Stiborová et al., 2014b). On the other hand, under aerobic conditions AAI acts as a classical substrate of CYP1A1 or 1A2, and takes one atom of atmospheric oxygen to *O*-demethylate the methoxy group of AAI to generate AAIa. In line with this suggestion is the finding that binding of AAI to the active site of the Compounds I of CYP1A1 and 1A2 indeed favors *O*-demethylation of AAI to AAIa (see Fig. 5 in Stiborová et al., 2015b). However, as shown in Fig. 2, the increased reductive activation of AAI *ex vivo* had no apparent impact on the reductive metabolism of AAI *in vivo*; AAI-DNA adduct formation was attenuated by induction of CYP1A enzymes. Likewise, induction of cytosolic NQO1, which led to an increase in AAI-DNA adduct formation *ex vivo*, had no significant effect *in vivo*, as a decrease in AAI-DNA adduct levels was observed. These findings demonstrate that *in vivo* the oxygen concentrations in rat tissues are sufficient to facilitate the process of the oxidative *O*-demethylation of AAI, which is thereafter the predominant reaction of CYP1A1/2 in AAI metabolism *in vivo*. Therefore, in addition to the influence of CYP1A expression, the *in vivo* pO_2_ in tissues is an important factor that affects the balance between nitroreduction and *O*-demethylation of AAI, thereby influencing its (geno) toxicity and carcinogenicity. Indeed, the presence of oxygen in the *in-vitro* incubations of AAI with DNA and microsomal or cytosolic enzymes strongly inhibits the levels of AAI-DNA adducts formed in these systems (Schmeiser et al., 1997).

Based on the present study and taking into account previous results obtained in *Cyp1a*-knock-out and *CYP1A*-humanized mouse lines (Rosenquist et al., 2010, Arlt et al., 2011, Stiborová et al., 2012, Stiborová et al., 2014a, Stiborová et al., 2014b, Stiborová et al., 2014c), we conclude that the efficiency of the CYP1A family to protectively oxidize AAI to AAIa prevails over its reducing activation *in vivo*. The evaluation of inter-individual variations in the human CYP1A enzymes, including their genetic polymorphisms, remains a major challenge to explain human individual susceptibility to AAI, and to predict the risk of cancer among patients suffering from AAN and BEN.

## Conflict of interest

The authors declare that there are no conflicts of interest.

## Funding

Financial support from Grant Agency of the Czech Republic (grant 14-18344S) and Charles University in Prague (grants UNCE 204025/2012 and 570513) is highly acknowledged. Work at King’s College London is supported by Cancer Research UK (grant C313/A14329).

## Figures and Tables

**Table 1:** Relative expression of mRNA of hepatic, renal and pulmonary CYP1A1, CYP1A2 and NQO1 in liver, kidney and lung from untreated (control) animals and animals treated with AAI, Sudan I or AAI combined with Sudan I.

		Liver		Kidney		Lung	
		Δ*c*_T_^a^	Fold change over control	Δ*c*_T_^a^	Fold change over control	Δ*c*_T_^a^	Fold change over control
*CYP1A1*	Control	12.84 ± 0.44	*1.00*	7.22 ± 0.22	*1.00*	15.20 ± 0.15	*1.00*
	AAI	9.93 ± 0.44	7.54***	7.56 ± 0.27	0.791	13.53 ± 0.29	3.19**
	Sudan I	0.36 ± 0.06	5710***	4.53 ± 0.35	6.45***	2.08 ± 0.04	8930***
	Sudan I + AAI	1.56 ± 0.31	2490***	4.53 ± 0.17	6.63***	2.05 ± 0.22	9090***
*CYP1A2*	Control	0.75 ± 0.34	*1.00*	16.38 ± 0.42	*1.00*	19.89 ± 0.18	*1.00*
	AAI	−2.22 ± 0.08	7.86***	14.60 ± 0.32	3.43**	12.26 ± 0.26	198***
	Sudan I	−5.23 ± 0.44	63.2***	7.85 ± 0.25	370***	7.30 ± 0.26	6170***
	Sudan I + AAI	−5.76 ± 0.16	91.5***	8.72 ± 0.82	202***	10.67 ± 0.43	595***
*NQO1*	Control	6.03 ± 0.24	*1.00*	7.51 ± 0.16	*1.00*	5.98 ± 0.46	*1.00*
	AAI	2.10 ± 0.29	15.2***	7.27 ± 0.18	1.18	5.66 ± 0.27	1.25
	Sudan I	1.06 ± 0.22	31.2***	5.88 ± 0.28	3.10**	2.97 ± 0.08	8.06***
	Sudan I + AAI	1.47 ± 0.28	23.5***	6.05 ± 0.31	2.76**	3.42 ± 0.44	5.92***

a Values relative to β-actin are means ± S.D. from data found for three male rats (*n* = 3) (control and treated with AAI, Sudan I and AAI with Sudan I). The induction of mRNA expression of studied target genes in treated animals was evaluated as 2^−(ΔΔcT)^ (see Section 2). Comparison was performed by Student’s *t*-test analysis.

** *P* > 0.01.

*** *P* > 0.001 significantly different from controls.
