# Supplementary material for: Induction of cytochromes P450 1A1 and 1A2 suppresses formation of DNA adducts by carcinogenic aristolochic acid I in rats in vivo
Source: Toxicology. 2016 Feb 17;344-346:7–18. doi: 10.1016/j.tox.2016.01.011 (PMC4804751; doi:10.1016/j.tox.2016.01.011)
Supplement: Supplementary file 1 [file mmc1.doc]

**Supplementary Table 1.** AAI-DNA adduct formation in livers, kidneys and lungs of rats treated with AAI, Sudan I and AAI after pretreatment with Sudan I

**A**

________________________________________________________________________

Organ RAL^a^/10^8^

dG-AAI dA-AAI dA-AAII Total

__________________________________________________________________________

**Liver**

Control (untreated) rats No AAI-DNA adducts

Rats treated with Sudan I No AAI-DNA adducts

Rats treated with AAI 0.80 ± 0.18 5.40 ± 0.47 0.96 ± 0.31 7.16 ± 0.67

Rats treated with Sudan I

and AAI 0.42 ± 0.10^***^ 1.90 ± 0.27^***^ 0.60 ± 0.25^***^  2.92 ± 0.51^***^

(**F =** **0.40 fold**)

**Kidney**

Control rats No AAI-DNA adducts

Rats treated with Sudan I No AAI-DNA adducts

Rats treated with AAI 1.04 ± 0.23 2.91 ± 0.25 0.62 ± 0.19 4.57 ± 0.71

Rats treated with Sudan I

and AAI 0.50 ± 0.07^***^ 1.29 ± 0.31^***^ 0.25 ± 0.08^***^ 2.04 ± 0.29^***^

(**F = 0.45 fold**)

**Lung**

Control (untreated) rats No AAI-DNA adducts

Rats treated with Sudan I No AAI-DNA adducts

Rats treated with AAI 0.42 ± 0.01 1.83 ± 0.38 0.28 ± 0.03 2.51 ± 0.42

Rats treated with Sudan I

and AAI 0.28 ± 0.05^***^ 0.78 ± 0.15^***^ 0.21 ± 0.06^***^ 1.27 ± 0.57^***^

(**F = 0.50 fold**)

___________________________________________________________________________

Values are given as the means ± SD (*n* = 3); each DNA sample was determined by two postlabeled analyzes. Comparison was performed by *t*-test analysis; **P* < 0.001, different from animals treated with AAI alone. “F” indicates fold changes in DNA adduct levels in animals treated with Sudan I and AAI compared to animals treated with AAI alone.

^a^Relative adduct labeling.

**Supplementary Figure 1.** HPLC chromatogram of AAI oxidation metabolites formed by hepatic microsomes of control rats incubated with AAI and NADPH. HPLC was carried our with an Nucleosil 100-5 C_18_, 250 × 4.0 mm, 5 mm (Macherey-Nagel) column, using a linear gradient of acetonitrile (20 to 60% acetonitrile in 55 min) in 100 mM triethylamonium acetate with a flow rate of 0.6 ml/min. A Dionex HPLC pump P580 with UV/VIS UVD 170S/340S spectrophotometer detector set at 254 nm was used. Peaks were integrated with CHROMELEON™ 6.01 integrator. A peak eluting at retention time (r.t.) 24.5 min was identified as AAIa using mass-spectroscopy analysis (Levová et al., 2011).
